# BRCA1 Interacting Protein COBRA1 Facilitates Adaptation to Castrate-Resistant Growth Conditions

**DOI:** 10.3390/ijms19072104

**Published:** 2018-07-20

**Authors:** Huiyoung Yun, Roble Bedolla, Aaron Horning, Rong Li, Huai-Chin Chiang, Tim-H Huang, Robert Reddick, Aria F. Olumi, Rita Ghosh, Addanki P. Kumar

**Affiliations:** 1 Department of Urology, The University of Texas Health, San Antonio, TX 78229, USA; Huiyoung_Yun@DFCI.HARVARD.EDU (H.Y.); BEDOLLAR@uthscsa.edu (R.B.); ghoshr@uthscsa.edu (R.G.); 2 Pharmacology, the University of Texas Health, San Antonio, TX 78229, USA; 3 Molecular Medicine, The University of Texas Health, San Antonio, TX 78229, USA; Horning@livemail.uthscsa.edu (A.H.); lir3@uthscsa.edu (R.L.); chiangh@uthscsa.edu (H.-C.C.); huangt3@uthscsa.edu (T.-H.H.); 4 UT Health San Antonio Cancer Center, the University of Texas Health, San Antonio, TX 78229, USA; 5 Pathology, the University of Texas Health, San Antonio, TX 78229, USA; REDDICK@uthscsa.edu; 6 Department of Urology, Massachusetts General Hospital Harvard Medical School, 55 Fruit Street, Yawkey Building, Suite 7E, Boston, MA 02215, USA; AOLUMI@PARTNERS.ORG; 7 South Texas Veterans Health Care System, San Antonio, TX 78229, USA

**Keywords:** COBRA1, NELFB, androgen receptor, CRPC

## Abstract

COBRA1 (co-factor of BRCA1) is one of the four subunits of the negative elongation factor originally identified as a BRCA1-interacting protein. Here, we provide first-time evidence for the oncogenic role of COBRA1 in prostate pathogenesis. COBRA1 is aberrantly expressed in prostate tumors. It positively influences androgen receptor (AR) target gene expression and promoter activity. Depletion of COBRA1 leads to decreased cell viability, proliferation, and anchorage-independent growth in prostate cancer cell lines. Conversely, overexpression of COBRA1 significantly increases cell viability, proliferation, and anchorage-independent growth over the higher basal levels. Remarkably, AR-positive androgen dependent (LNCaP) cells overexpressing COBRA1 survive under androgen-deprivation conditions. Remarkably, treatment of prostate cancer cells with well-studied antitumorigenic agent, 2-methoxyestradiol (2-ME_2_), caused significant DNA methylation changes in 3255 genes including COBRA1. Furthermore, treatment of prostate cancer cells with 2-ME_2_ downregulates COBRA1 and inhibition of prostate tumors in TRAMP (transgenic adenocarcinomas of mouse prostate) animals with 2-ME_2_ was also associated with decreased COBRA1 levels. These observations implicate a novel role for COBRA1 in progression to CRPC and suggest that COBRA1 downregulation has therapeutic potential.

## 1. Introduction

Prostate cancer (PCA) continues to be the second leading cause of cancer related deaths in men, with the overwhelming majority of deaths due to castration resistant prostate cancer (CRPC) [1]. Given that early stage PCA is dependent on androgen receptor (AR) signaling, androgen-deprivation therapy (ADT) is the standard therapeutic approach for clinical management of PCA. While ADT is effective in regressing tumor growth, the response is transient (12–18 months) leading to cancer relapse. Relapse of cancer following ADT (known as CRPC) occurs because the recurring tumors grow either in the absence of or low concentrations of androgens [2,3]. CRPC is fatal as no effective durable systemic therapy currently exists. Despite castrate levels of androgens, AR signaling is still active under these conditions and human prostate tumors express AR. Reactivation of AR signaling occurs through numerous mechanisms, such as AR amplification, mutation, splice variants, coregulators, inflammatory cytokines, and receptor tyrosine kinases, contribute to the development of CRPC [4]. These data suggest that development and progression of CRPC is complex and involves compensatory signaling networks. Thus, understanding the molecular factors that contribute to progression to CRPC is critical for successful clinical management of PCA. Towards achieving this goal, we identified an unexpected role for activation of cofactor of BRCA1 (COBRA1), a protein traditionally known to be involved in transcription pausing as yet another mechanism potentially contributing to progression to aggressive prostate cancer.

COBRA1 (aka NELF-B) is one of the four subunits of the negative elongation regulatory (NELF) complex originally identified as a BRCA1-interacting protein. COBRA1 prevents transcriptional elongation by stalling RNA polymerase II (RNAPII) at the proximal promoter region [5,6]. Given its ability to repress transcriptional activity of multiple oncogenes such as estrogen receptor alpha (ERα), earlier studies suggested that COBRA1 may play a tumor-suppressor role [7,8]. Emerging evidence suggests paradoxical oncogenic and tumor suppressive roles for COBRA1. For example, human gastrointestinal adenocarcinomas show increased COBRA1 expression and protein levels compared to normal upper gastrointestinal tract implicating a potential oncogenic role for COBRA1 [9]. Recent studies also indicate a developmental role since mouse embryonic fibroblasts from COBRA1-KO animals show reduced proliferation and elevated apoptosis [10,11]. Interestingly, COBRA1 functions as an AR co-activator by virtue of its ability to interact with AR ligand-binding domain (LBD) [12]. Clinically, patients carrying BRCA mutations are at significantly elevated risk for developing metastatic disease and death from PCA [13]. Furthermore, recent studies showed that nearly 20% of prostate cancer patients who carry the BRCA1 biallelic mutation are at risk for developing castrate resistant prostate cancer. More importantly, these patients carrying biallelic inactivation of BRCA2 are responsive to PARP-1 inhibitors further emphasizing the clinical relevance [14]. Recent mouse genetic studies strongly suggest a mutually antagonistic role of COBRA1 and BRCA1 in both mammary gland development and mammary tumorigenesis [15,16]. However, the role of COBRA1 in prostate cancer is largely unknown. Based on these evidence, we tested whether COBRA1 plays a role in prostate pathogenesis either directly or through its regulatory effects on gene expression. Here, we provide first time evidence for an oncogenic role for COBRA1 in human prostate cancer and its potential as a therapeutic target.

## 2. Results and Discussion

**Figure 1:**
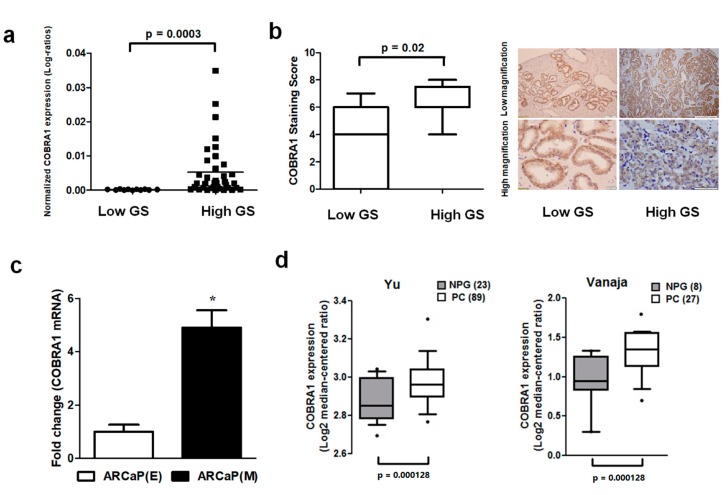
Expression of COBRA1 in human prostate tumors: (**a**) Expression of COBRA1 as assessed by qRT-PCR using the Tissue Scan Prostate Cancer Tissue qPCR Panel III (Origene, Rockville, MD, USA) comprising of low (GS < 7; *n* = 11) and high (GS ≥ 7; *n* = 37) GS tumors. Data was analyzed using unpaired two-tailed *t*-test with Welch’s correction (*p* = 0.0003); (**b**) Immunohistochemical evaluation of COBRA1 in human prostate tumor microarray comprising low (GS < 7; *n* = 11) and high (GS ≥ 7; *n* = 13) GS tumors. Cumulative analysis of this data is presented as box plot. Statistical analysis of the data was performed using unpaired two-tailed *t*-test with Welch’s correction; (**c**) COBRA1 mRNA expression was measured by qRT-PCR in ARCaP (E) and ARCaP (M) cells. Error bars indicate ±S.E.M. (*n* = 3). * *p* < 0.05; (**d**) Box plots of COBRA1 expression in normal prostate gland (NPG) and prostate carcinoma (PC) from Oncomine database (http://www.oncomine.org, accessed on 19 May 2015). Data sets are log transformed and illustrated as median centered box plots between the differences of mRNA expression within cohorts. Statistical significance was determined by a two-tailed Mann–Whitney test. IHC pictures shown are at 100 and 500 microns (low magnification images) and 100 and 20 microns (high magnification images) for low and high GS tumors respectively.

Basal level and expression of COBRA1 were analyzed in a panel of human prostate cancer cell lines, human prostate tumor array comprising of low (<7) and high (≥7) Gleason score (GS) tumors and a commercial cDNA prostate tissue array. We observed (i) elevated mRNA expression of COBRA1 with increasing tumor aggressiveness (Figure 1a); (ii) significantly increased COBRA1 protein levels in high GS tumors compared with low GS tumors (Figure 1b); and (iii) elevated levels of COBRA1 in an advanced mesenchymal phenotype cell line compared with its isogenic epithelial counterpart (Figure 1c). In silico analysis of Oncomine data showed significantly elevated mRNA expression in prostate tumors compared to normal prostate gland (Figure 1d). These data taken together suggest a potential role for COBRA1 in prostate cancer progression.

**Figure 2:**
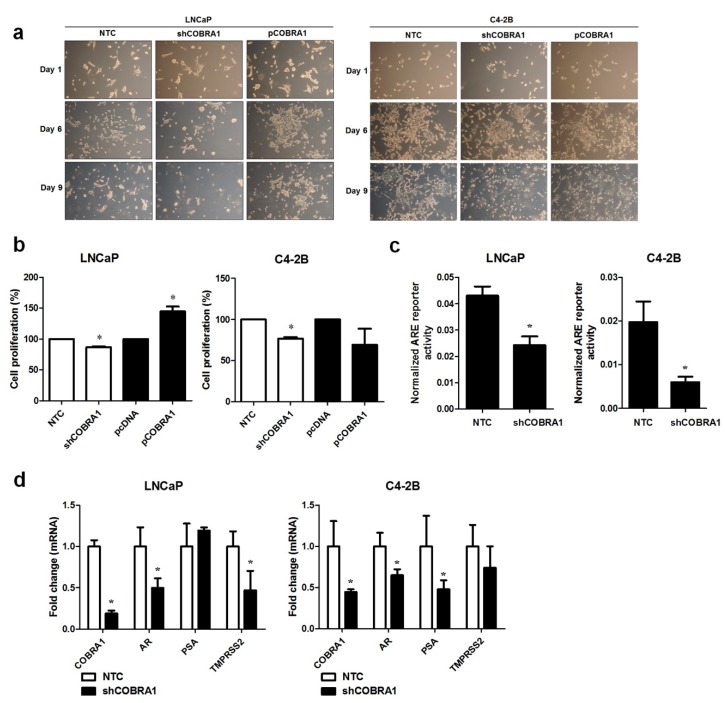
COBRA1 facilitates progression to castrate resistance: (**a**) Androgen responsive LNCaP and its castrate resistant sub line C4-2B stably silenced or ectopically expressing COBRA1 with respective controls were grown in charcoal stripped media for 10 days. Cells were observed microscopically for any morphological changes. A representative image at 10× magnification from three independent experiments is shown; (**b**) Proliferative ability of androgen responsive LNCaP and its castrate resistant sub line C4-2B stably silenced or ectopically expressing COBRA1. Data presented is an average of three independent experiments conducted in triplicate. Error bars indicate ±S.E.M. (*n* = 3). * *p* < 0.05; (**c**) ARE reporter activity in androgen responsive LNCaP and its castrate resistant sub line C4-2B stably silenced for COBRA1. Data presented is an average of three independent experiments conducted in triplicate. Error bars represent ±S.E.M. (*n* = 3). * *p* < 0.05; (**d**) mRNA expression changes of AR, PSA, and TMPRSS2 in androgen responsive LNCaP and its castrate resistant sub line C4-2B stably silenced for COBRA1. Data presented is an average of three independent experiments conducted in triplicate. Error bars indicate ± S.E.M. (*n* = 3). * *p* < 0.05.

It was previously shown that COBRA1 interacts with AR LBD and can function as a coactivator of AR [12]. This evidence led to the hypothesis that COBRA1 facilitates androgen independency. To investigate this proposition, AR expressing androgen responsive LNCaP and castrate resistant C4-2B cells with COBRA1 knockdown and overexpression were grown under androgen-deprived conditions. Vector transfected (NTC) and COBRA1 silenced LNCaP cells (shCOBRA1) failed to thrive under these conditions; while COBRA1 overexpressing (pCOBRA1) cells formed large colonies and thrived under androgen-deprived conditions (Figure 2a, left panel). Surprisingly, overexpression or knockdown of COBRA1 had no effect on growth of C4-2B cells under these experimental conditions (Figure 2a, right panel). Stable knockdown of COBRA1 in LNCaP and C4-2B was accompanied by a small but significant reduction in proliferation under hormone-replete conditions (Figure 2b) and COBRA1 overexpression enhanced proliferation in LNCaP but not in C4-2B cells (Figure 2b). These results taken together suggest that COBRA1 may be involved in cellular adaptation under castrated conditions but may not be an important player after cells have adapted to grow in the absence of androgens. To investigate if COBRA1 activates AR signaling, we analyzed AR reporter activity and mRNA expression changes in AR and its bonafide target genes, PSA and TMPRSS2. COBRA1 silenced LNCaP and C4-2B cells had significantly reduced AR-reporter activity in both LNCaP and C4-2B cells (Figure 2c). While silencing COBRA1 significantly reduced AR message levels in LNCaP and C4-2B cells; the AR target genes affected differed between the 2 cells lines. In LNCaP cells TMPRSS2 was significantly reduced while in C4-2B cells PSA (prostate-specific antigen) was significantly reduced (Figure 2d) suggesting differential participation of COBRA1 in AR-mediated transcriptional regulation between androgen-responsive and castrate resistant cells. However, whether subtle differences in the amount of COBRA1 knockdown contributes to the observed differences cannot be ruled out. We interpret these observations to suggest that COBRA1 expression facilitates progression to castrate resistant disease by affecting AR signaling. Our results do not rule out the role for other nuclear receptors in mediating these effects. Further, COBRA1 can physically interact with other transcription factors including Sp1 or Sp3 as there is precedence for interaction of COBRA1 with c-Fos and AP-1 [17]. Our study sets the stage for additional work to understand the mechanism(s) of COBRA1 involvement in prostate cancer progression.

**Figure 3:**
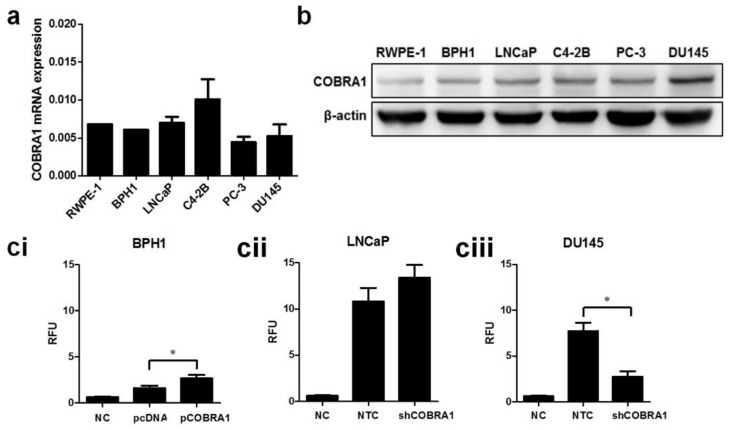

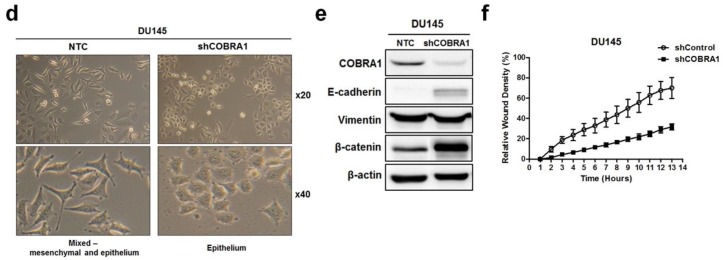
Involvement of COBRA1 in epithelial plasticity: (**a**,**b**) Total RNA and whole cell lysates prepared from logarithmically growing RWPE-1, BPH1, LNCaP, C4-2B, PC-3, and DU145 was used in measuring mRNA expression and protein levels of COBRA1 respectively. In immunoblot analysis β-actin was used as loading control; (**c**) Anchorage-independent growth assay in (**ci**) nontumorigenic BPH1 cells ectopically expressing COBRA1 (pCOBRA1), vector control (pcDNA), or negative control without cells (NC); (**cii**) LNCaP cells and (**ciii**) DU145 cells silenced for COBRA1 (shCOBRA1) or a scrambled shRNA (NTC). Data presented is an average of three independent experiments conducted in triplicate. Statistical analysis of the data was calculated using student’s t-test. Error bars indicate ±S.D. (*n* = 3). * *p* < 0.05; (**d**) Logarithmically growing androgen independent DU145 stably silenced COBRA1 cells with respective controls were observed microscopically for morphological changes. Representative image is shown; (**e**) Immunoblot analysis of E-cadherin, Vimentin and β-catenin in DU145 cells silenced for COBRA1. A representative immunoblot from three independent experiments is shown; (**f**) Migratory ability of DU145 cells silenced for COBRA1. Data shown is a representative of three independent experiments. Error bars indicate ± S.D. (*n* = 3).

There was no significant difference in COBRA1 message among nontransformed and various prostate cancer cell lines although protein level was higher in cancer cells compared with nontransformed cells (Figure 3a,b). Based on protein levels we chose to examine the biological effects of COBRA1 modulation using BPH1 (overexpression), LNCaP, and DU145 (silencing) cells. We observed consistent overexpression (~2 fold) in BPH1-C (BPH1-COBRA1) cells and ~0.5 fold knockdown of COBRA1 in LNCaP and DU145 cells (Figure S1a). Overexpression of COBRA1 resulted in enhanced anchorage independent growth in BPH1 cells, while silencing COBRA1 resulted in decreased anchorage independent growth in DU145 (highest basal COBRA1 level) and had no significant change in LNCaP cells (Figure 3ci–ciii). It is noteworthy to mention that although BPH1 cells exhibited significant increase in anchorage-independent growth, these cells grew slower than the cancer cells, perhaps an indication of their nontumorigenic nature. Similar effects were observed on cell viability with COBRA1 modulation (Figure S1b).

Examination of the morphology of DU145-shCOBRA1 cells showed distinct changes suggestive of epithelial phenotype compared with the non-targeted shRNA transfected cells (NTC) cells that appeared to have a mix of mesenchymal and epithelial phenotype (Figure 3d). This observation prompted us to examine the proteins that are well established markers of epithelial–mesenchymal transition (EMT). We found increased levels of E-cadherin and β-catenin with no changes in vimentin (Figure 3e). These observations are consistent with the data presented in Figure 1c showing that ARCaP-M (mesenchymal cells) have significantly higher expression of COBRA1 than ARCaP-E (epithelial) cells. These data lead us to believe that high levels of COBRA1 in DU145 cells may be associated with cell plasticity due to the lack of E-cadherin/β-catenin complex that play important roles in epithelial barrier. Since gain of cell migration and loss of cell adhesion is a characteristic of mesenchymal cells, we used real-time cell imaging migration assay to test whether COBRA1 silencing would affect the migratory capability. We found significantly decreased migration of shCOBRA1-DU145 cells as a function of time compared with the NTC cells (Figure 3f).

**Figure 4:**
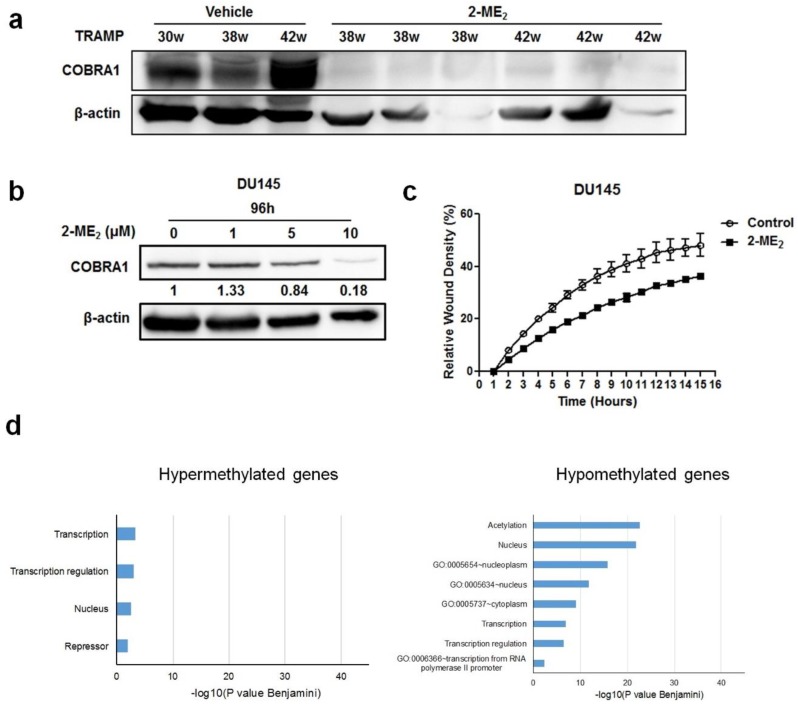
Pharmacological inhibition of COBRA1 reduces migratory ability of DU145 cells and prostate tumor progression: (**a**) Western blot analysis of COBRA1 in the prostate from TRAMP treated with or without 2-ME_2_; (**b**) Western blot analysis of COBRA1 in the DU145 cells treated with or without 2-ME_2_ (1, 5 and 10 µM) for 24 h; (**c**) Migratory ability of DU145 cells treated with or without 2-ME_2_ (5 µM). Data shown is a representative of three independent experiments. Error bars indicate ± S.D. (*n* = 3); (**d**) DNA methylation changes to COBRA1-related transcription-regulation pathways are affected by 2-ME_2_ in a concentration dependent manner. Statistically significant gene ontology term associations are indicated by bars ≥1.3.

The data presented thus far shows that COBRA1 is overexpressed in prostate tumors and contributes to the adaptation and survival of prostate cancer cells under castrate conditions. To examine whether COBRA1 could serve as a therapeutic target, we analyzed protein changes in the prostate and tumors samples obtained from a retrospective 2-ME_2_ intervention study conducted in transgenic adenocarcinoma of the mouse prostate (TRAMP) model. We previously demonstrated that 2-ME_2_ (i) intervention regressed prostate tumor growth in this model and (ii) down regulates c-FLIP [18,19,20]. Analyses of COBRA1 protein levels showed significant decrease in the prostate from 2-ME_2_ intervention group compared to the vehicle control (Figure 4a). Consistent with these in vivo observations, treatment with 2-ME_2_ decreased COBRA1 protein levels in DU145 cells in a dose-dependent manner (Figure 4b). 5 µM 2-ME_2_ treatment decreased migration of DU145 cells significantly as a function of time (Figure 4c). These results suggest that 2-ME_2_ could suppress migratory ability of prostate cancer cells in part via inhibition of COBRA1. Furthermore, treating castrate resistant C4-2B cells with 2-ME_2_ (3 µM) caused significant (*p* < 0.05) DNA methylation changes in 3,255 genes (*n* = 91 hypermethylated and *n* = 3164 hypomethylated) including COBRA1 according to results obtained with an Infinium HumanMethylation450 BeadChip Kit. Functional annotation charts using the Database for Annotation and Visualization and Integrated Discovery (DAVID) on the 3000 most hypomethylated (negative fold change) genes revealed pathways associated with transcription and transcriptional regulation (Figure 4d). For the 1 µM treatment, only the genes exhibiting hypermethylation were run in DAVID together whereas for the 3 µM treatment, only the genes exhibiting hypomethylation were run in DAVID together. Bar charts indicating the level of significance of the association of the DAVID ontology terms with each treatment groups’ list of differentially methylated genes (Figure 4d). Of note, hypermethylated genes produced the chart for cells treated with 1 µM 2-ME_2_ because almost all of the methylation changes observed were positive fold changes. Conversely, treatment with 3 µM 2-ME_2_ produced hypomethylated genes because most of the methylation changes observed in this treatment group were negative fold changes. Interestingly, we identified COBRA1 as one of the hypomethylated genes in these pathways. Although comprehensive investigations are necessary to conclude whether 2-ME_2_-mediated decreased expression in COBRA1 is indeed due to changes in its methylation status, nonetheless, these results suggest that 2-ME_2_ suppresses prostate tumorigenesis possibly by altering the methylation status of COBRA1. This could explain many of the previous observations regarding changes in gene expression in response to 2-ME_2_ observed by various groups. While this study does not demonstrate the involvement of 2-ME_2_ in transcriptional pausing, it would be interesting to test the hypothesis that 2-ME_2_ inhibits COBRA1-mediated RNA Pol II transcriptional activity to prevent prostate pathogenesis. While this manuscript was under preparation, an oncogenic role for the negative elongation factor E (NELFE) was identified in hepatocellular carcinoma [21].

Although localized prostate cancer can be effectively treated, options for treatment of metastatic castrate resistant disease (CRPC) are mostly palliative with no cure and is therefore a major clinical challenge. Although the treatment landscape for management of CRPC has changed significantly over the past decade, still the pathways that activate AR signaling in the absence or low levels of androgens is poorly defined. These observations underscore the need to understand the cellular, biochemical, and molecular alterations associated with pathological progression to castrate resistance. Along these lines, data presented in this manuscript that show COBRA1 as a potential factor contributing to progression to castrate resistance are significant. To the best of our knowledge these data for the first time implicate oncogenic role of COBRA1 in prostate cancer progression through its ability to allow adaptation to castrate-resistant growth conditions and the loss of epithelial barrier integrity. We also provide evidence that COBRA1 may be a novel therapeutic target in prostate cancer management since treatment with anti-estrogenic compound(s) inhibits COBRA1-related effects observed in prostate cancer. Furthermore, emerging evidence links germline and somatic mutations in DNA repair genes including BRCA1 with castrate resistance [22]. Given that COBRA1 is a BRCA1 interacting protein, we speculate that therapeutic targeting of COBRA1 could provide an additional option for patients with DNA repair aberrations.

## 3. Materials and Methods

### 3.1. Cell Culture and Reagents

AR-positive androgen dependent (LNCaP), AR-negative androgen independent (PC-3 and DU145) human prostate cancer cells were purchased from American Type Culture Collection (Manassas, VA, USA), and AR-positive androgen independent (C4-2B) were obtained from Dr. Thambi Dorai (Department of Biochemistry and Molecular Biology, New York Medical College, Valhalla, NY, USA), and benign prostate cells (BPH1) were obtained from Dr. M.S. Lucia (Department of Pathology, University of Colorado Denver, Denver, CO, USA). BPH1, LNCaP, C4-2B, and DU145 cells were maintained in RPMI-1640 media supplemented with 10% fetal bovine serum (FBS) and 1% penicillin/streptomycin in a humidified incubator supplied with 5% CO_2_ and at 37 °C. PC-3 cells were grown in F12-K media containing 10% FBS plus antibiotics. Logarithmically growing LNCaP, PC-3, C4-2B (3 µM), and DU145 (5 µM) cells were treated with 2-methoxyestradiol (2-ME_2_) (Sigma, St. Louis, MO, USA).

### 3.2. COBRA1 Stable Cell Generation

COBRA1 stable knockdown cells were generated with shRNA targeting COBRA1 using pSUPER-retro-neo retroviral shRNA expression plasmid (Oligoengine, Seattle, WA, USA). In parallel, control cells were generated using a scrambled shRNA. The optimal concentration of G418 (neomycin) for selection and maintenance of COBRA1 stable cells was established by performing kill curve using range of G418 concentrations (0.1–2.0 mg/mL). Cells were seeded at a density of 1 × 10^5^ cells/mL in complete media in T75 flask. Following their attachment, cells were transfected with 10 μg of total plasmid DNA per flask using Lipofectamine 2000 reagent (15 μL; Invitrogen, Grand Island, NY, USA). G418 selection was used to select transfected cells 48–72 h post-transfection (BPH1, 1000 μg/mL; LNCaP, C4-2B, DU145, 500 μg/mL). G418 was replaced every 2–3 days by adding fresh media containing appropriate dose of G418, and cells were examined visually for toxicity daily. Cells were maintained in the media containing G418 and collected as a polyclonal line. The polyclonal cells were plated sparsely at a very low density (~10 cells/well) in 6-well plate and allowed to form individual colonies. The individual colonies were trypsinized and transferred to 10 cm dish for monoclonal expansion. The efficiency of COBRA1 overexpression or knockdown was verified using western blotting and qRT-PCR. However, we noted that the knockdown efficiency decreases with time. For ectopic expression, cells were transfected with pcDNA3.1-based expression vectors for COBRA1 or mock transfected with the empty vector.

### 3.3. Western Blot and Quantitative Real Time PCR

Whole-cell extracts were prepared using 2× SDS buffer supplemented with fresh protease and phosphatase inhibitors. Equal volumes of protein were fractionated by SDS-PAGE and transferred to nitrocellulose membranes. The primary antibodies used include anti-COBRA1, 1:1000, (Dr. Rong Li, Department of Molecular Medicine, University of Texas Health San Antonio, San Antonio, TX, USA), β-actin, 1:2000, (Sigma, St. Louis, MO, USA), E-cadherin, 1:1000, (Cell signaling, Danvers, MA, USA), Vimentin, 1:1000, (Cell signaling), β-catenin, 1:2000, (Cell signaling). Bound antibody was visualized using ECL kit (Thermo Fisher Scientific, Waltham, MA, USA). All the blots were stripped and reprobed with β-actin to ensure equal loading of protein. Images were captured and analyzed using Gene snap software (Syngene, Frederick, MD, USA), and quantification was carried out using Gene tools software (Syngene).

Total cellular RNA was isolated using Trizol reagent (Invitrogen). RNA was reverse transcribed using the SuperScript VILO cDNA Synthesis Kit (Invitrogen). Target genes were amplified and expression was measured using 7300 Applied Biosystems with SYBR Green dye. The qRT-PCR was conducted with the primers as follows:

β-actin,forward 5’-GGCACCCAGCACAATGAAGATCAA-3’
reverse 5′-TAGAAGCATTTGCGGTGGACGATG-3′COBRA1,forward 5′-GTTCCAGACAGAGAATGGTG-3′
reverse 5′-ATACCGACTGGTGGAACT-3′AR, forward 5′-AGGAGGAAGGAGAGGCTTCC-3′
reverse 5′-GAGCAAGGCTGCAAAGGAGT-3′TMPRSS2, forward 5′-TACTCTGGAAGTTCATGGGCAGCA-3′
reverse 5′-AAGTTTGGTCCGTAGAGGCGAACA-3′PSA,forward 5′-AATCGATTCCTCAGGCCAGGTGAT-3′
reverse 5′-AGAACTCCTCTGGTTCAATGCTGC-3′

PCR reactions were conducted in triplicate, and relative mRNA expression was normalized to β-actin. Fold change in experiments was determined relative to solvent control group. Specific amplification of target genes was validated using a dissociation curve.

### 3.4. Luciferase Assay

For transfections, human prostate cancer cells were plated in triplicate at a density of 1 × 10^5^ cells per well in 24-well plates. Following their attachment, cells were transfected with ARE reporter plasmids (0.5 μg) along with Renilla luciferase (10 ng) using Lipofectamine 2000 reagent (Invitrogen,). Luciferase activity was determined after 36 h transfection using the Dual Luciferase Reporter Assay system (Promega, Madison, WI, USA) essentially as described previously [20].

### 3.5. Cell Growth and Proliferation

Trypan blue, soft agar growth, and MTT assays were used to determine growth and survival. For trypan blue assay, cells were plated at a density of 1 × 10^4^ cells/well in 24-well plates for 2–3 days, and then trypsinized, combined with the Trypan blue reagent (Sigma, St. Louis, MO, USA), and cell numbers were counted. For soft agar assay, cells were seeded at a density of 10,000 cells/well in 96 well plate containing semisolid agar media. Transformation ability of these cells was measured using CytoSelect^TM^ 96-well Cell Transformation Assay (Cell Biolabs, San Diego, CA, USA) following 6–8 days incubation. Fluorescence was read on SpectraMax M5 plate reader (Molecular Devices, San Jose, CA, USA) using 485/520 nm. Briefly, cells growing in semisolid agar media were solubilized, lysed, and incubated with CyQuant GR Dye (Cell Biolabs) for measuring fluorescence. For cell proliferation, cells were seeded in triplicate at a density of 4 × 10^3^ per well in 96-well plate. Cell proliferation was detected following 72 h incubation essentially as described previously [20] by measuring absorbance at 570/650 nm.

### 3.6. Migration Assay

The migration rate of androgen independent prostate cancer cells was assessed using the real-time cell imaging system (IncuCyte^TM^ live-cell ESSEN BioScience Inc., Ann Arbor, MI, USA). A scratch was made using the 96-pin WoundMaker^TM^ (ESSEN BioScience Inc.) in cells growing in 96 well plate. Cell migration was monitored in real time over a period of 14 h, and images were automatically acquired and analyzed using IncuCyte^TM^ 96-well Cell Migration Software Application Module (ESSEN BioScience Inc.). Data is represented as the Relative Wound Density (RWD), which is a representation of the spatial cell density in the wound area relative to the spatial cell density outside of the wound area at every time point (time-curve).

### 3.7. DNA Methylation Array

Global DNA methylation levels in androgen independent prostate cancer cells C4-2B were measured following treatment with 2-ME_2_ (1 μM and 3 μM) for 5 days. By using a MethylMiner Methylated DNA enrichment kit (Invitrogen), methylated DNA was isolated from fragmented whole genomic DNA via binding to the methyl-CpG binding domain of human MBD protein coupled to paramagnetic Dynabeads M-280 Streptavidin through a biotin linker. Then, samples were subjected to DNA methylation analysis on the Illumina HumanMethylation450 Beadchip Kit (BASIC core facility at UTHSA). This analysis produced a list of genes with significant changes in DNA methylation (hyper or hypomethylation; *p* < 0.05). For each gene list, up to 3000 genes were selected and run in the Database for Annotation and Visualization and Integrated Discovery (DAVID) that produced Functional Annotation Charts.

### 3.8. Animal Experiments

Western blot analysis of COBRA1 levels were examined in TRAMP (transgenic adenocarcinomas of mouse prostate), prostate tumors and tissues were obtained from a repository available from previous studies in our laboratory [18,19]. Tissues were procured from a study testing the potential of 2-ME_2_ (50 mg/kg body weight through drinking water) by administrating to 22–25 week old of TRAMP mice for additional 25 weeks [18,19]. Proteins were extracted using RIPA buffer from the tumors and tissues in the control group (30, 38, and 42 weeks), and in the treatment group (38 and 42 weeks).

### 3.9. Immunohistochemistry

COBRA1 rabbit polyclonal antibody (Dr. Rong Li, Department of Molecular Medicine, University of Texas Health San Antonio, San Antonio, TX, USA) was used. Sections from paraffin embedded tissues were heat cleared and rehydrated. Antigen retrieval was performed with citrate buffer at pH 6.0 in a 121 °C pressure chamber. Endogenous peroxidase was quenched with a TBS buffer containing 3% hydrogen peroxide followed by a protein blocking buffer incubation. Each step was carried out at room temperature. The sections were incubated for 1 h at room temperature with the antibody. The negative control sections were incubated with a Universal Rabbit negative control Rabbit Ig fraction (DAKO Corp., Carpinteria, CA, USA). The ancillary and visualization systems were: Rabbit HRP polymer (BioCare Medical, Concord, CA, USA) and DAB Chromogen System (DAKO Corp.). IHC slides were evaluated and graded by pathologist (R. R) in a blinded fashion. The total COBRA1 staining was scored as the product of the staining intensity (on a scale of 0–3) and the percentage of cells stained (on a scale of 0–5), resulting in a scale of 0–8. Staining intensity was scored as follows: 0, none of the cells scored positively; 1, weak staining; 2, moderate staining intensity; and 3, strong staining intensity. Percent staining was scored as follows: 1, 20%; 2, 30%; 3, 60%; 4, 80% and 5, 100% cells stained. Low (100 μm) and high (50 μm) magnification images were taken using a Nikon Eclipse Ci microscope equipped with camera (D5-F12).

### 3.10. Oncomine Data

COBRA1 expressed in normal prostate gland and prostate carcinoma were obtained from two independent studies for each gene expression in the Oncomine database. Primary sources are from different group’s microarray data mentioned in the graph (http://www.oncomine.org). Data sets are log transformed and illustrated as median centered box plots between the differences of mRNA transcription within cohorts. Statistical significance was determined by a two-tailed Mann–Whitney test. Detailed information of the standardized normalization and statistical calculations are indicated on the Oncomine website.

### 3.11. Statistical Analysis

All numerical results are expressed as mean ± S.D. or S.E.M. derived from 3 independent experiments, unless otherwise stated. Statistical analyses were conducted using Student’s *t*-test and statistically significant differences were established as *p* < 0.05. The statistical significance of IHC data was calculated using unpaired two-tailed t test with a Welch’s correction.

## Supplementary Materials

Supplementary materials can be found at http://www.mdpi.com/1422-0067/19/7/2104/s1.

## Abbreviations

COBRA1	Co-factor of BRCA1
2-ME_2_	2-methoxyestradiol
TRAMP	Transgenic adenocarcinoma of the mouse prostate
PCA	Prostate cancer
CRPC	Castration resistant prostate cancer
AR	Androgen receptor
ADT	Androgen-deprivation therapy
NELF	Negative elongation factor
RNAPII	RNA polymerase II
ERα	Estrogen receptor alpha
LBD	Ligand binding domain
EMT	Epithelial-mesenchymal transition
DAVID	Database for annotation and visualization and integrated discovery
