# Supplementary material for: BRCA1 Interacting Protein COBRA1 Facilitates Adaptation to Castrate-Resistant Growth Conditions
Source: Int J Mol Sci. 2018 Jul 20;19(7):2104. doi: 10.3390/ijms19072104 (PMC6073349; doi:10.3390/ijms19072104)
Supplement: Supplementary file 1 [file ijms-19-02104-s001.zip › ijms-317364-SI.pdf]

International Journal of
Molecular Sciences

Supplementary

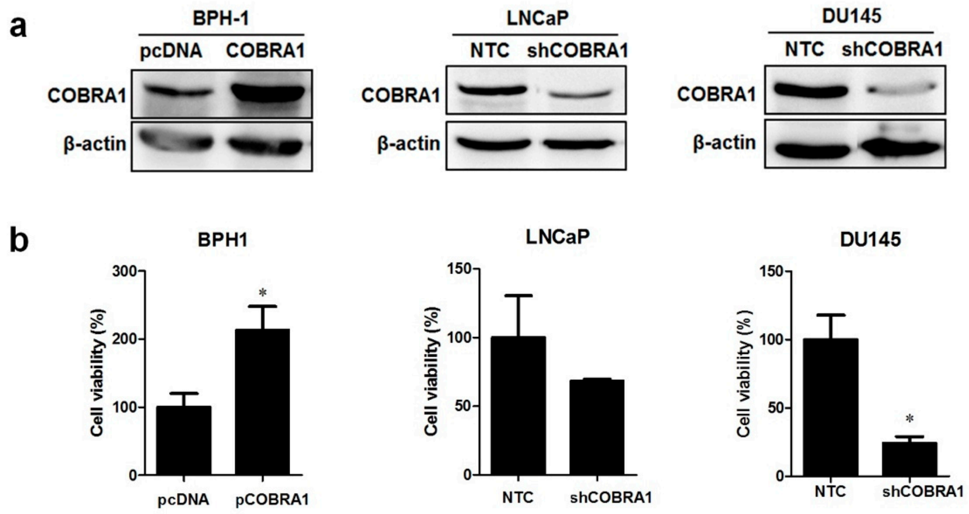

Figure S1. (**a**) Validation of COBRA1 overexpression or knockdown efficiency; (**b**) Cell viability was assessed by trypan blue staining. Data shown is a representative of three independent experiments. Error bars indicate ± S.D. ($n = 3$).
